# Cardiovascular disease burden in patients with urological cancers: The new discipline of uro‐cardio‐oncology

**DOI:** 10.1002/cai2.108

**Published:** 2024-02-05

**Authors:** Yi Zheng, Ying Liu, Ziliang Chen, Yunpeng Zhang, Zuo Qi, Ning Wu, Zhiqiang Zhao, Gary Tse, Yong Wang, Hailong Hu, Yuanjie Niu, Tong Liu

**Affiliations:** ^1^ Tianjin Key Laboratory of Ionic‐Molecular Function of Cardiovascular Disease, Department of Cardiology, Tianjin Institute of Cardiology Second Hospital of Tianjin Medical University Tianjin China; ^2^ School of Nursing and Health Studies Hong Kong Metropolitan University Hong Kong China

**Keywords:** anticancer therapy, cardiovascular toxicity, mechanism, multidisciplinary management, urological cancer

## Abstract

Cancer remains a major cause of mortality worldwide, and urological cancers are the most common cancers among men. Several therapeutic agents have been used to treat urological cancer, leading to improved survival for patients. However, this has been accompanied by an increase in the frequency of survivors with cardiovascular complications caused by anticancer medications. Here, we propose the novel discipline of uro‐cardio‐oncology, an evolving subspecialty focused on the complex interactions between cardiovascular disease and urological cancer. In this comprehensive review, we discuss the various cardiovascular toxicities induced by different classes of antineoplastic agents used to treat urological cancers, including androgen deprivation therapy, vascular endothelial growth factor receptor tyrosine kinase inhibitors, immune checkpoint inhibitors, and chemotherapeutics. In addition, we discuss possible mechanisms underlying the cardiovascular toxicity associated with anticancer therapy and outline strategies for the surveillance, diagnosis, and effective management of cardiovascular complications. Finally, we provide an analysis of future perspectives in this emerging specialty, identifying areas in need of further research.

## Graphical abstract

Treatment‐induced cardiovascular toxicity is linked to ADT, VEGFR‐TKIs, ICIs, and chemotherapy. We propose the new discipline of uro‐cardio‐oncology to address the relationship between cardiovascular disease and urological cancers. ADT, androgen deprivation therapy; ICI, immune checkpoint inhibitor; VEGFR‐TKI, vascular endothelial growth factor receptor tyrosine kinase inhibitor.

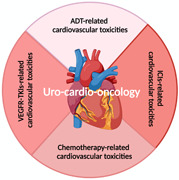

**Abbreviations**
ADT: androgen deprivation therapy
CTLA‐4: cytotoxic T lymphocyte‐associated antigen‐4
GnRH: gonadotropin‐releasing hormone
ICI: immune checkpoint inhibitor
PD‐1: programmed cell death protein 1
PD‐L1: programmed cell death protein ligand 1
RCC: renal cell carcinoma
TKI: tyrosine kinase inhibitor
VEGFR: vascular endothelial growth factor receptor

## 1 BACKGROUND

Cardiovascular disease is highly prevalent, and the associated morbidity, mortality, and healthcare costs have led to its recognition as a significant public health concern with global implications. Cardiovascular diseases have consistently been among the leading causes of death worldwide [1]. The top three risk factors for cardiovascular disease are hypertension, an unhealthy diet, and elevated low‐density lipoprotein cholesterol levels [2]. Among the various types of cardiovascular disease, ischemic heart disease is the foremost cause of death, responsible for a staggering 9.44 million fatalities in 2021 alone [3]. As such, efforts to address this issue remain a critical priority for public health initiatives worldwide.

Urological cancers are the most common group of cancers affecting men. Based on epidemiological data, urological cancer alone contributed to almost 41% of new cancer diagnoses among men in the United States in 2023 [4]. Several therapeutic agents have been used to treat urological cancer, improving the prognosis for patients. Nevertheless, there is a rising incidence of cardiovascular complications caused by antineoplastic therapy. Late morbidity and mortality are predominantly caused by cardiovascular diseases among cancer survivors [5], and the presence of treatment‐induced cardiovascular toxicity is notably linked with prostate cancer [6], renal cell carcinoma (RCC) [7], and bladder cancer [8], as well as other urological cancers.

To address the relationship between cardiovascular disease and urological cancer, we propose a new discipline, uro‐cardio‐oncology. The present review aims to provide a comprehensive summary of the cardiovascular toxicities associated with urological cancer therapy, along with strategies for the surveillance, diagnosis, and management of cardiovascular complications. We conclude this article by highlighting areas in need of further research in this emerging specialty.

## 2 ANDROGEN DEPRIVATION THERAPY‐RELATED CARDIOVASCULAR TOXICITY

Prostate cancer is the most common malignancy in men [9]. Risk factors include advanced age, a family history of cancer, racial and ethnic background, and a sedentary lifestyle [10]. Prostate cancer is a hormone‐driven adenocarcinoma, with tumor proliferation chiefly regulated by androgens. Androgen deprivation therapy (ADT) is the mainstay of prostate cancer treatment, with approximately 40% of patients receiving neoadjuvant or adjuvant ADT with radiotherapy or receiving treatment following biochemical relapse after prostate cancer surgery [11]. Patients with prostate cancer are at risk of noncancer mortality due to the development of cardiovascular disease. Extensive observational data have indicated that men who receive ADT are more likely to experience hypertension, ischemic heart disease, and cancer therapy‐related cardiac dysfunction [12, 13]. A large randomized controlled trial quantified long‐term adverse events among patients with prostate cancer receiving intermittent ADT versus continuous ADT, reporting a 10‐year cumulative incidence of ischemic or thrombotic events of 33% for intermittent ADT and 24% for continuous ADT (hazard ratio [HR] = 0.69, *p* = 0.02) [14]. While ADT is not frequently associated with QTc prolongation on an electrocardiogram, in rare cases, it can result in torsade de pointes due to the blocking of testosterone effects on ventricular repolarization [15, 16]. These findings highlight the importance of carefully monitoring cardiovascular risks in patients receiving ADT. Event rates between different types of ADT (medical castration, bilateral orchiectomy, and both medical castration and bilateral orchiectomy) were comparable.

Gonadotropin‐releasing hormone (GnRH) agonists and antagonists are key components of ADT. The comparative cardiovascular safety of GnRH agonists versus antagonists in prostate cancer remains controversial. An international randomized clinical trial prospectively compared the cardiovascular safety of degarelix, a GnRH antagonist, versus leuprolide, a GnRH agonist, in patients with prostate cancer [17]. Over a 1‐year follow‐up, the incidence of major adverse cardiovascular events did not differ significantly between patients who were randomized to receive degarelix or leuprolide (HR = 1.96; 95% confidence interval [CI]: 0.59–2.79; *p* = 0.53). While most patients with metastatic prostate cancer currently opt for a GnRH agonist as the preferred ADT, recent evidence suggests that use of GnRH antagonists may reduce the risk of cardiovascular events. The HERO phase III clinical trial conducted a comparative analysis between relugolix, a GnRH antagonist, and leuprolide in patients with advanced prostate cancer [18]. Despite a numerically greater and more sustained suppression of testosterone levels over 48 weeks with relugolix, the incidence of major adverse cardiovascular events, including central nervous system hemorrhage, myocardial infarction, and cerebrovascular conditions, was significantly lower with relugolix compared with leuprolide. In contrast, recent real‐world population‐based data from public hospitals in Hong Kong showed that GnRH antagonists may be associated with greater long‐term, but not short‐term, cardiovascular risks than agonists in Asian patients with prostate cancer, particularly in those without known cardiovascular risk factors [19]. Additionally, the duration of GnRH agonist use is positively associated with cardiovascular risk, and thus, future work should investigate the optimum duration [20].

The correlation between cardiovascular events and use of neoadjuvant ADT before radiation therapy appears to be influenced by pre‐existing cardiovascular disease. In patients with prostate cancer receiving ADT, the presence of heart failure, myocardial infarction, or arrhythmia alone, but not stroke, was associated with raised cardiovascular risk. Interestingly, while cardiovascular risk factors were increasingly prevalent in patients with prostate cancer receiving ADT, the risk of major adverse cardiovascular events also increased despite decreasing mortality [21]. Specifically, evidence suggests that a history of myocardial infarction or ischemic congestive heart failure may be indicative of an elevated risk of all‐cause mortality with ADT (HR = 1.96; 95% CI: 1.04–3.71) [22]. In patients with metastatic prostate cancer, those with a history of two or more prior cardiovascular events had the highest probability of experiencing additional cardiovascular events following initiation of ADT (HR = 1.91; 95% CI: 1.66–2.20) [6]. It is noteworthy that approximately one‐third of patients diagnosed with metastatic prostate cancer have a history of cardiovascular disease when ADT is initiated. These findings are in agreement with data from Hong Kong, where the number of major cardiovascular comorbidities was more important prognostically than the type of comorbidity in patients with at least two of the following: heart failure, myocardial infarction, stroke, and arrhythmia [23]. Hence, considering pre‐existing cardiovascular risk factors and their influence on treatment outcomes is integral to optimal prostate cancer management.

The above reports have heightened attention to and discussion of the metabolic implications of ADT and the potential correlation between ADT and increased cardiovascular risk. The elevated risk may be caused by metabolic changes induced by ADT, including hyperglycemia and dyslipidemia [24]. Results from a multicenter randomized clinical trial indicate that ADT may elevate the risk of cardiovascular disease through weight gain, reduced insulin sensitivity, and dyslipidemia [25]. Moreover, among patients with advanced prostate cancer receiving ADT with a GnRH agonist, a randomized controlled trial reported significant lean body mass reductions of 0.76 kg (95% CI: −1.53–0.00; *p* = 0.015) and fat mass increases of 2.95 kg (95% CI: 2.16–3.74; *p* < 0.001) after 48 weeks of treatment [26]. Notably, ADT appears to promote the accumulation of subcutaneous rather than visceral fat [27, 28]. In addition, ADT has been observed to increase serum cholesterol and triglyceride levels. In a randomized controlled trial of 519 patients with prostate cancer, the group treated with ADT had significant increases in serum total cholesterol, low‐density lipoprotein cholesterol, high‐density lipoprotein cholesterol, and triglycerides [29]. Most of these changes occurred within the first few months of treatment initiation, highlighting the need for early lipid monitoring in patients receiving ADT. Some studies have suggested that hyperinsulinemia may be an independent risk factor for the development of cardiovascular disease [30, 31]. ADT has been observed to increase fasting plasma insulin in men with prostate cancer, which is considered a marker of insulin resistance [32]. In a 3‐month prospective study involving men without diabetes, ADT administration resulted in a statistically significant 26% increase in fasting plasma insulin levels and a 13% decrease in insulin sensitivity [33]. The long‐term effects of ADT on insulin sensitivity need further investigation. Other metabolic risk factors include raised uric acid levels [34]. Finally, in a subgroup analysis of patients with prostate cancer receiving ADT who also had diabetes, visit‐to‐visit variability in HbA1c was a significant prognostic marker [35], and metformin use was protective against adverse cardiovascular outcomes and mortality [36, 37].

## 3 VASCULAR ENDOTHELIAL GROWTH FACTOR RECEPTOR TYROSINE KINASE INHIBITOR (VEGFR‐TKI)‐RELATED CARDIOVASCULAR TOXICITY

Five VEGFR‐TKIs have been approved by the FDA for RCC, namely sunitinib, pazopanib, axitinib, sorafenib, and cabozantinib [38, 39]. These agents target intracellular regions of the VEGFR, thereby modulating the survival of microvascular endothelial cells [40]. VEGFR inhibitors also elicit increased migration of T lymphocytes toward neoplastic tissue, thereby augmenting the sensitivity of malignant cells to immunotherapeutic interventions [41]. Encouraging clinical outcomes were observed in randomized phase III trials evaluating VEGFR‐TKIs for metastatic RCC, and the efficacy of both pazopanib and sunitinib has been confirmed by real‐world evidence; these two drugs are currently the most used in good‐ and intermediate‐risk patients with metastatic RCC [42]. However, research increasingly suggests that despite their anticancer benefits, VEGFR‐TKIs can be cardiotoxic.

Sunitinib is an oral multitargeted TKI. Its use has been linked to increased cardiovascular risk, attributable to alterations in blood supply, cellular metabolism, signal transduction, and transcription [7]. Sunitinib has been associated with several cardiovascular complications, including increased serum creatine kinase levels (at a rate of 49%), elevated blood pressure (15%–39%), peripheral edema (up to 24%), decreased left ventricular ejection fraction (11%–16%), chest pain (13%), venous thromboembolism (4%), and heart failure (3%). Notably, hypertensive crisis and hemorrhage are severe complications that may prove life‐threatening [43]. Thus, patients receiving sunitinib treatment should have their blood pressure and ejection fraction monitored closely. Pazopanib is an inhibitor of VEGFR 1–3 and therefore inhibits angiogenesis and promotes RCC regression [44]. Hypertension is the most common cardiovascular toxicity associated with pazopanib, with an incidence of 40%–42%. Other side effects include cardiac arrhythmias (bradycardia [2%–19%], QT prolongation [<2%], torsades de pointes [<1%]), peripheral edema (14%), heart failure (11%–13%), thromboembolism (5%), ischemic heart diseases (≤2%), and myocardial infarction (≤2%) [45]. Of the other VEGFR‐TKIs, sorafenib is a multikinase inhibitor that can hamper tumor growth [46]. Axitinib is well tolerated from a cardiovascular standpoint, with hypertension as the most common side effect [47]. Cabozantinib is a highly efficacious multitargeted TKI with activity against both mesenchymal–epithelial transition factor and VEGFR. Cabozantinib treatment can lead to a notable increase in both progression‐free survival and objective response rates. A randomized, open‐label, phase III trial reported that cabozantinib‐related cardiotoxicity included hypertension (30%–61%), hypotension (7%), thrombotic events (1%–7%), and vascular disease (1%) [48].

## 4 IMMUNE CHECKPOINT INHIBITOR (ICI)‐RELATED CARDIOVASCULAR TOXICITY

ICIs, such as ipilimumab, nivolumab, pembrolizumab, and avelumab, have been approved for treating metastatic RCC. These ICIs have two distinct mechanisms: heightened T‐cell priming via blockade of cytotoxic T lymphocyte‐associated antigen‐4 (CTLA‐4) or activation of the tumor immune response by blocking programmed cell death protein 1 (PD‐1) or its ligand, programmed cell death protein ligand 1 (PD‐L1) [49]. Cardiovascular toxicity, including heart failure, cardiomyopathy, myocardial fibrosis, conduction abnormalities, myocarditis, pericarditis, vasculitis, arrhythmias, myocardial infarction, and cardiac arrest, has been reported in patients with RCC treated with ICIs [50, 51]. The prevalence of cardiovascular adverse events is higher when ICIs are used in combination therapy. In the first‐line treatment setting for metastatic RCC, two therapy options can be considered: dual ICI therapy with nivolumab and ipilimumab or a combination of targeted therapy and ICI therapy, such as axitinib plus avelumab or pembrolizumab, cabozantinib plus nivolumab, or lenvatinib plus pembrolizumab [52]. Compared with monotherapy, dual ICI therapy is associated with higher rates of autoimmune toxicity [53]. Autoimmune myocarditis has received substantial attention in recent literature, although its reported incidence has been relatively low, ranging from 0.27% to 1.14%. However, despite various management strategies being proposed, this toxicity remains highly lethal [54]. Targeted therapies for RCC have considerably different patterns of toxicity compared with ICIs. VEGF inhibitors have the potential to induce a reduction in left ventricular ejection fraction, although this may be relatively infrequent. A randomized, open‐label phase III trial reported the potential for overlapping toxicities with cardiovascular toxicity such as colitis and hepatitis [55]. Compared with dual ICI therapy, targeted therapy in combination with an ICI has higher overall response rates. In addition, relevant data showed that cabozantinib combined with nivolumab provided quality of life benefits [55]. The safety of the combination is yet to be confirmed in further clinical studies.

## 5 CHEMOTHERAPY‐RELATED CARDIOVASCULAR TOXICITY

Bladder cancer is the most common malignant tumor affecting the urinary system; it shows a progressive rise in incidence over time and is particularly prevalent in the expanding aging population [56]. Cigarette smoking and occupational exposure are widely recognized as the principal risk factors [57]. In recent years, focus on the cardiovascular toxicity linked to chemotherapy regimens for bladder cancer has increased. Perfusion chemotherapy has been discussed as a potential therapeutic strategy. This innovative modality entails local delivery of chemotherapeutic agents into the bladder, facilitating precise and concentrated drug distribution at the tumor site. This aims to optimize treatment effectiveness while mitigating systemic adverse effects. Multiple studies have shown encouraging results with improvements in tumor response rates and overall survival [58, 59]. However, anthracycline chemotherapy‐related cardiac dysfunction can manifest clinically or be identified in asymptomatic individuals during surveillance [60]. Therefore, close cardiac monitoring is recommended in patients who receive anthracycline chemotherapy.

Cisplatin has generally favorable cardiovascular tolerability. Although the incidence of cisplatin‐induced cardiotoxicity is rare (<1%), sporadic cases have been documented [61, 62]. Clinical presentations include cardiovascular abnormalities such as arrhythmias, cardiomyopathy, myocarditis, and congestive heart failure [8, 61].

Gemcitabine, a nucleoside analog, is extensively used as monotherapy or in combination regimens for various malignancies [63]. According to the FDA, gemcitabine does not have significant cardiotoxicity. It is commonly used in elderly or frail patients due to its relatively favorable toxicity profile compared with other anticancer medications. However, an international global pharmacovigilance study showed that gemcitabine was associated with several cardiovascular adverse drug reactions, including myocardial ischemia, supraventricular arrhythmias, and heart failure [63]. In the event of cardiotoxicity, discontinuation of gemcitabine is recommended.

## 6 POSSIBLE MECHANISMS OF CARDIOVASCULAR TOXICITY RELATED TO ANTICANCER THERAPY

### 6.1 ADT

The effects of ADT on the pathogenesis and progression of atherosclerotic cardiovascular disease have traditionally been attributed to ADT‐induced metabolic disturbances, which potentially have atherogenic consequences. Obesity, dyslipidemia, and diabetes mellitus are widely recognized as risk factors for atherosclerotic cardiovascular disease. Additionally, emerging evidence suggests that visceral adiposity, hyperglycemia, elevated low‐density lipoprotein levels, and reduced high‐density lipoprotein levels are associated with the development of atherosclerotic plaques [64]. Frequently, these metabolic disturbances are concomitant, giving rise to metabolic syndrome. ADT may exacerbate these metabolic dysfunctions. A recent meta‐analysis showed that patients with prostate cancer who underwent ADT faced a 75% increased risk of developing incident metabolic syndrome compared with those who did not (relative risk 1.75, 95% CI: 1.27–2.41) [65]. Similarly, in a cohort study involving 12,191 patients with localized prostate cancer, ADT use was correlated with a 60% elevated risk of incident diabetes mellitus (HR = 1.61; 95% CI: 1.38–1.88) [66]. The mechanism underlying ADT‐induced type 2 diabetes has been elucidated in murine hepatocytes; knockout of androgen receptors enhanced the activation of gluconeogenic pathways while attenuating the activation of glycolytic pathways [67].

ADT may also increase cardiovascular risk via testosterone reduction. Several animal studies have established an association between androgen deprivation and the progression of atherosclerosis, while others have demonstrated the advantageous effects of testosterone using mouse models of atherosclerotic disease [68, 69]. Experimental evidence has shown that testosterone has atheroprotective properties, including the attenuation of monocyte adhesion and transmigration across the vascular endothelium [70]. Reduced levels of testosterone have also been linked to an elevated risk of major adverse cardiovascular events [71].

**Figure 1:**
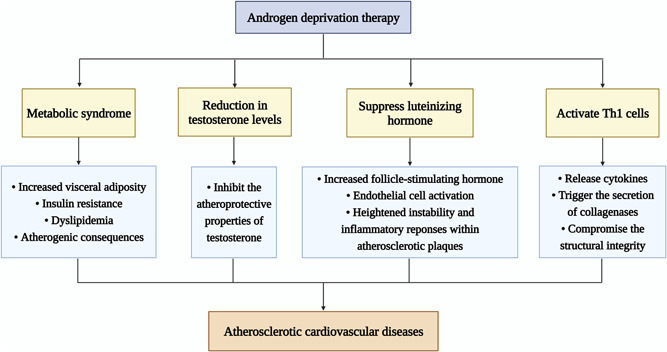
Mechanisms of androgen deprivation therapy leading to atherosclerotic cardiovascular disease.

As discussed in Section 2, GnRH agonists have been associated with a higher risk of cardiovascular toxicity compared with GnRH antagonists, potentially attributable to their suppression of luteinizing hormone. This suppression may lead to an increase in follicle‐stimulating hormone, which binds to its receptors on vascular endothelial cells. Subsequent endothelial cell activation may lead to heightened instability and inflammatory responses within atherosclerotic plaques [72]. Furthermore, administration of GnRH agonists has been shown to activate type 1 T helper cells, leading to the release of pro‐inflammatory cytokines. This cascade of events subsequently triggers collagenase secretion, compromising the structural integrity of pre‐existing atherosclerotic plaques and rendering them more susceptible to rupture [72]. The mechanisms leading to ADT‐induced atherosclerotic cardiovascular diseases are shown in Figure 1.

### 6.2 VEGFR‐TKIs

VEGF inhibitors modulate multiple signaling pathways [60]. Associated cardiovascular toxicity may be due to the inhibition of tyrosine kinases expressed in the blood vessels and myocardium. Since VEGF signaling is crucial for maintaining cardiac function, adverse effects may result from the inhibition of this signaling [73]. VEGF plays an important role in the proliferation and survival of endothelial cells and contributes to the integrity of the vascular system. Inhibiting VEGF signaling has been shown to diminish the regenerative capacity of endothelial cells, increase smooth muscle proliferation, and increase hematocrit and blood viscosity, which are associated with pro‐coagulant changes and subsequent thrombosis [73, 74]. In the absence of VEGF signaling, oxidative stress, glomerular injury, vascular rarefaction, and inhibition of the nitric oxide pathway can occur. This may result in hypertension and vascular disease [75]. Additionally, VEGF inhibition can lead to renal thrombotic microangiopathy. According to available data, sequestration of VEGF may lead to impaired adaptive cardiac hypertrophy because of pressure overload [76].

Evidence of sunitinib‐associated cardiovascular toxicity is the most well established [7, 77]. Sunitinib is associated with endothelin‐1 system activation, coronary microvascular dysfunction, adenosine 5ʹ‐monophosphate‐activated protein kinase inhibition leading to mitochondrial dysfunction, abnormal energy homeostasis in cardiomyocytes, as well as inhibition of the receptor for mast/stem cell growth factor [78]. One study confirmed this finding by incubating rat neonatal cardiomyocytes with high‐dose sunitinib. Sunitinib was found to activate a caspase‐9‐associated mitochondrial apoptotic pathway and reduce both mitochondrial membrane potential and energy levels as a result of adenosine 5ʹ‐monophosphate‐activated protein kinase activity inhibition [79]. In addition, sunitinib's effects on adenosine 5ʹ‐monophosphate‐activated protein kinase and platelet‐derived growth factor receptors may influence cardiomyocyte function and survival [80].

### 6.3 ICIs

The broad application of ICI therapy has led to the detection of ICI‐associated cardiovascular side effects, among which myocarditis is the most well recognized. Blockade of CTLA‐4 signaling disturbs the equilibrium of the immune system, causing spontaneous activation of T cells and acute myocardial damage. Ying et al. [81] found that experimental autoimmune myocarditis in wild‐type mice was increased when CTLA‐4‐B7 interaction was blocked in vivo. Similarly, another study showed that mice lacking CTLA‐4 developed severe myocarditis and pancreatitis because of lymphoproliferative disease accompanied by multiorgan lymphocytic infiltration [82].

The interaction between PD‐1 and PD‐L1 in the heart reduces inflammation‐related myocyte damage by downregulating the local immune response [83]. As reported by Lucas et al. [84], PD‐L1‐deficient MRL mice developed lethal lymphocytic myocarditis marked by massive infiltration of CD8+ and CD4+ T cells. By these findings, it has been assumed that ICIs interfere with CTLA‐4 and PD‐1 signaling, lowering the threshold for T‐cell activation and breaking down peripheral immune tolerance. Additionally, anti‐CTLA‐4 monoclonal antibodies may reduce the number of regulatory T cells that constitutively express CTLA‐4, resulting in enhanced activation of T cells that are highly reactive to cardiac stimuli [85].

A second theory suggests that ICI‐associated myocarditis could be caused by the clonal expansion of T cells able to recognize the same homologous antigen present in both the tumor and the myocardium. Researchers discovered that T‐cell receptors from tumor and myocardial T cells were identical when they sequenced these receptors from next‐generation T cells [86]. These findings suggest that T cells may target epitopes common to the myocardium and tumor and that ICIs can enhance T‐cell effector function, leading to autoimmune myocarditis.

### 6.4 Chemotherapy

**Figure 2:**
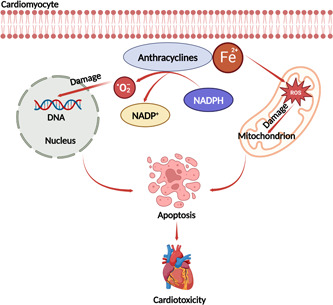
Mechanisms of anthracycline‐induced cardiac toxicity. Anthracyclines can bind to Fe^2+^ in cardiomyocytes, leading to the generation of ROS, further oxidative stress, and subsequent mitochondrial dysfunction. Fe^2+^ forms a complex with anthracyclines that generates free radicals via NADPH oxidase, which can damage DNA. The combination of DNA damage, oxidative stress, and mitochondrial dysfunction can induce apoptosis in cardiomyocytes, leading to cardiotoxicity. Fe^2+^, ferrous iron; NADPH, nicotinamide adenine dinucleotide phosphate; O_2_^−^, superoxide anion; ROS, reactive oxygen species.

The chemotherapies most likely to induce cardiovascular toxicity are anthracyclines and fluoropyrimidines. The mechanisms associated with anthracycline‐related cardiotoxicity are multifactorial [87]. Release of free radicals, mitochondrial dysfunction, abnormalities in intracellular calcium handling, and/or iron homeostasis can all cause damage to the heart [88]. Of these mechanisms, the free radical‐mediated damage pathway is the most well known. Reduction of the anthracycline quinone group produces a semiquinone group, which is rapidly oxidized to form superoxide‐producing hydrogen peroxide. Subsequently, hydrogen peroxide interacts with the myocardium. Ferric iron in the body can form complexes with anthracyclines and generate more free radicals. After combining, ferrous iron is converted to ferric iron, which damages the mitochondrial, nuclear membranes, the endoplasmic reticulum, and cell membranes, leading to a decrease in intracellular calcium ions and a reduction in cardiac contractility [89, 90] (Figure 2).

The key pathophysiologic processes of 5‐fluorouracil‐related cardiotoxicity include coronary vasospasm and endothelial injury [91]. Animal experiments showed that rabbits treated with 5‐fluorouracil developed severe endothelial damage, platelet accumulation, and fibrin formation [92]. Thrombosis may be triggered after vessel damage. In addition, anthracyclines can cause paradoxical vasoconstriction due to endothelial dysfunction or abnormal endothelial nitric oxide synthase. Thus, the chronic vasoconstriction associated with 5‐fluorouracil may be cardiotoxic [93].

## 7 CARDIOVASCULAR SURVEILLANCE, DIAGNOSIS, AND MANAGEMENT IN PATIENTS WITH UROLOGICAL CANCER RECEIVING ANTICANCER TREATMENT

### 7.1 Cardiovascular surveillance

Thorough clinical evaluation and physical examination are recommended to detect any early signs or symptoms of cardiovascular complications when cancer treatment is administered. Patients with cancer may be at increased risk of developing cardiac arrhythmias, particularly those undergoing chemotherapy or radiation therapy [94, 95, 96]. Since these arrhythmias can lead to serious health complications, including heart failure, stroke, and sudden cardiac arrest, [97, 98] ECG monitoring is required for all at‐risk patients and may also be necessary for patients who have pre‐existing heart conditions or have a family history of heart disease [99]. Regular ECG monitoring facilitates early detection of cardiac abnormalities, enabling appropriate medical intervention.

Serum cardiac biomarkers indicate the presence of heart injury or damage. These biomarkers can be used for cardiovascular toxicity screening during anticancer therapy and may also help guide therapy [100]. Consequently, they have become a valuable tool in the screening and management of cardiac toxicity in patients with cancer, allowing doctors to significantly reduce the risk of cardiac toxicity and improve survival outcomes for patients [101, 102]. Natriuretic peptides and cardiac troponin should be used for cancer therapy‐related cardiac dysfunction screening and diagnosis [103, 104]. Early detection allows early intervention by healthcare professionals, preventing further damage to the heart. However, since cancer treatments have different effects on the release of natriuretic peptide and cardiac troponin, increased biomarker levels should be interpreted within the context of the patient's clinical condition [105]. Notably, generally accepted cut‐off or reference values for cardiovascular biomarkers in recipients of cancer treatment have yet to be defined. Additionally, many factors may affect natriuretic peptide and cardiac troponin levels, including age, sex, obesity, renal function, infection, and comorbidities like atrial fibrillation [105, 106].

Cardiac imaging is critical for clinical decision‐making during cancer treatment [107]. Cardiac magnetic resonance imaging and advanced echocardiography facilitate early detection and management of cardiovascular complications associated with anticancer therapy [108, 109]. Cardiac imaging should be performed promptly if patients present with cardiac symptoms. Transthoracic echocardiography is preferred to detect and diagnose cardiac dysfunction [110]. It is especially important to evaluate global longitudinal strain in cases of low‐normal left ventricular ejection fraction because it can determine whether there is asymptomatic myocardial damage [111, 112]. Thus, a relative change in global longitudinal strain has been suggested as the ideal tool to identify mild asymptomatic cancer therapy‐related cardiac dysfunction. The threshold for recommending a decrease in relative global longitudinal strain is currently 15% or more from baseline. This threshold allows more accurate diagnosis of cancer therapy‐related cardiac dysfunction and can guide selection of cardioprotective treatment [111]. Fast strain‐encoded cardiac magnetic resonance should be considered when transthoracic echocardiography image quality is poor or when transthoracic echocardiography does not provide diagnostic certainty [113, 114, 115].

### 7.2 Diagnosis and management of cardiovascular toxicity

Diagnosing cancer therapy‐related cardiac dysfunction can be challenging, as symptoms can be similar to those of other cardiovascular conditions, such as heart failure or arrhythmia. Patients may experience chest pain, shortness of breath, fatigue, or edema, among other symptoms [116]. Helpful investigations include ECG, echocardiography, cardiac magnetic resonance imaging, and cardiac biomarkers. Additionally, patients may undergo blood tests to evaluate cardiac biomarkers [117, 118]. Once a diagnosis is made, treatment options may include medications to improve cardiac function, such as beta‐blockers or angiotensin‐converting enzyme inhibitors, or diuretics to reduce fluid buildup in the body [119, 120]. A range of factors should be considered before starting treatment for cardiovascular symptoms, including current cancer and cardiovascular symptom burden, possible drug‐drug interactions, ongoing cancer treatment requirements, cancer prognosis, and patient preferences. In some cases, a reduction in the dose or frequency of cancer therapy may be necessary to reduce the risk of further cardiovascular damage. However, this decision must be carefully balanced with the need for effective anticancer treatment. In some cases, an alternative therapy that poses less risk to the heart can be considered [121]. Prevention of anticancer therapy‐related cardiovascular toxicity is also crucial. Patients at risk should be monitored closely during and after cancer treatment, especially those with pre‐existing cardiovascular disease. Additionally, lifestyle modifications, such as maintaining a healthy diet and exercise regimen, can help reduce the risk of cardiovascular complications. A coordinated multidisciplinary team is recommended for patients with cancer who develop acute cardiovascular complications. Patients with cancer must work closely with their healthcare team to manage both their cancer and cardiovascular health [122]. The diagnosis and management of cardiovascular toxicity is summarized in Table 1.

**Table 1:** The diagnosis and management of cardiovascular toxicities.

Cardiovascular toxicity	Classification	Diagnosis	Recommendations for management
CTRCD	Anthracycline chemotherapy‐related CTRCD	The presence of new cardiovascular symptoms, new abnormalities in cardiac function as observed through cardiovascular imaging, or newfound elevations in cardiac biomarkers	The decision to continue anticancer treatment is based on the severity of heart failure [108] Each case should undergo a thorough review by a multidisciplinary team for appropriate treatment
	Immune checkpoint inhibitor‐associated noninflammatory heart failure	Based on defining the heart failure phenotype and excluding myocarditis, takotsubo syndrome, and acute coronary syndrome [123]	
Arrhythmias	Atrial fibrillation	Clinical symptoms including palpitations, irregular heartbeats, shortness of breath, and fatigue Thorough physical examination Electrocardiography is the key diagnostic tool for confirming atrial fibrillation	Beta‐blockers are preferred among rate‐control drugs [124] For patients with heart failure/left ventricular dysfunction and/or uncontrolled symptoms, atrial fibrillation ablation should be considered [125]
	Long corrected QT interval and ventricular arrhythmias	Clinical evaluation Electrocardiography is crucial in diagnosing prolonged QTc and ventricular arrhythmias Additional tests may include exercise stress testing, 24‐h ambulatory electrocardiography monitoring, genetic testing to identify inherited forms of prolonged QT interval, and echocardiography to assess cardiac structure and function	Beta‐blockers are considered the optimal selection when the anticancer medication is also associated with CTRCD Amiodarone is the preferred pharmacological intervention for the treatment of arrhythmias in patients with haemodynamic instability and structural heart disease Decisions on the use of device therapy should consider complication risks, quality of life, and life expectancy [97]
	Bradyarrhythmias	Clinical evaluation, electrocardiography examination, and additional diagnostic tests	Intravenous injection of methylprednisolone is recommended for patients with PR prolongation >300 ms [126] For symptomatic patients, a Holter electrocardiogram is recommended
Arterial hypertension	Elevated hypertension Hypertension stage 1 Hypertension stage 2	120–129/<80 mmHg 130–139/80–89 mmHg ≥140/≥90 mmHg [127]	Angiotensin‐converting enzyme inhibitors/angiotensin receptor blockers are recommended as first‐line therapy of hypertension treatment to reduce the risk of CTRCD In patients with systolic blood pressure ≥160 mmHg and diastolic blood pressure ≥100 mmHg, combination therapy with an angiotensin‐converting enzyme inhibitor or angiotensin receptor blocker and a dihydropyridine calcium channel blocker is recommended [128]
PH	Pulmonary arterial hypertension PH associated with left heart disease PH associated with lung disease Chronic thromboembolic PH PH with unclear and/or multifactorial mechanisms	Echocardiography is the primary choice for evaluating PH probability The definitive diagnosis of PH and the subsequent determination of appropriate treatment strategies necessitate the utilization of right‐heart catheterization [129]	In patients who develop increase in peak tricuspid regurgitant velocity >3.4 m/s, implementation of right‐heart catheterization is advised Close monitoring of peak tricuspid regurgitant velocity with echocardiography should be considered in patients who manifest a novel asymptomatic peak tricuspid regurgitant velocity within the range of 2.9–3.4 m/s [129]
Coronary artery disease	Acute coronary syndromes	Symptoms, an early 12‐lead electrocardiogram, and serial assessments of high‐sensitivity cardiac troponin levels for patients exhibiting potential non‐ST‐segment elevation acute coronary syndromes	Temporary suspension of cancer treatment and expeditious adoption of a multidisciplinary approach are warranted to strategize personalized guideline‐driven management [122] In the absence of contraindications, initiation of suitable anti‐ischemic and antithrombotic therapy is recommended [122]
	Chronic coronary syndromes	The presence of stable angina, stress testing (exercise or pharmacological), imaging techniques such as coronary angiography, and measurement of biomarkers like troponin levels	Decisions on coronary revascularization should be made by a multidisciplinary team including experts in the fields of cardio‐oncology, interventional cardiology, and oncology The potential for heightened bleeding risk should be mitigated through the implementation of the shortest feasible duration of dual antiplatelet therapy [130]
Thromboembolic disease	Venous thromboembolism	Diagnosed through various methods such as ultrasound imaging to evaluate the presence of thrombus that form within the veins, particularly deep vein thrombosis and pulmonary embolism d‐dimer measurement can support the diagnosis of venous thromboembolism	Anticoagulant therapy is the cornerstone of VTE management Supportive measures involve pain management with analgesics and the use of compression stockings or intermittent pneumatic compression devices to prevent deep vein thrombosis and reduce swelling [131]
	Arterial thromboembolism	The diagnosis of arterial thromboembolism based on clinical evaluation, imaging examination, and assessment of risk factors	Prompt intervention to restore blood flow Invasive procedures are typically performed when there is a high risk of tissue damage or limb loss [132]
	Intracardiac thrombosis	Screening for cardiac etiology of thrombus formation initially with transthoracic echocardiography and/or transesophageal echocardiography Cardiac magnetic resonance imaging exhibits superior sensitivity and specificity compared to transthoracic echocardiography in the identification of intracardiac thrombi. Specifically, cardiac magnetic resonance utilizing the long inversion time technique for late gadolinium enhancement is presently considered as the gold standard [133]	Anticoagulant therapy is the mainstay of treatment In cases where there is a high risk of embolization or inadequate response to anticoagulation, invasive procedures may be necessary
Pericardial disease	Pericarditis Pericardial effusion	Characteristic changes in electrocardiography and echocardiography	In the absence of contraindications, treatment with anti‐inflammatory drugs and colchicine is recommended [134]
			In patients with signs of tamponade, expeditious percutaneous pericardiocentesis guided by echocardiography is the preferred approach Colchicine may confer clinical benefit in patients experiencing cardiac tamponade attributed to malignant pericardial effusions [135]
Valvular heart disease	Mitral valve disease Tricuspid valve disease Aortic valve disease	Echocardiography is the primary choice for the diagnosis of valvular heart disease	In patients with mechanical prosthetic valves, a meticulous assessment of the risk‐benefit ratio between thrombotic events and bleeding complications is warranted In patients presenting with significant valvular heart disease, a multidisciplinary team is required for the best treatment option
Immune checkpoint inhibitor‐associated myocarditis	Corticosteroid‐sensitive immune checkpoint inhibitor‐associated myocarditis Corticosteroid‐resistant immune checkpoint inhibitor‐associated myocarditis	Increase in troponin Electrocardiogram abnormalities Echocardiogram Magnetic resonance imaging Endomyocardial biopsy	Withdrawal of immune checkpoint inhibitor therapy Initiating high‐dose intravenous corticosteroids (methylprednisolone 500–1000 mg for 3d) [136]

Abbreviations: CTRCD, cancer therapy‐related cardiac dysfunction; PH, pulmonary hypertension.

## 8 FUTURE PERSPECTIVES ON THE EMERGING SPECIALTY

The future of uro‐cardio‐oncology is promising, driven by the increasing recognition of the complex interplay between cardiology, urology, and oncology. As highlighted in this review, several key areas warrant attention and exploration. First, further research efforts are needed to unravel the mechanisms underlying the development of cardiovascular complications in patients with urological cancers. By gaining a deeper understanding of these mechanisms, novel therapeutic targets can be identified and used to improve patient outcomes. This includes exploring relevant molecular pathways, genetic factors, and cellular processes involved in urological cancer‐related cardiovascular diseases. Additionally, the establishment of a consensus definition for uro‐cardio‐oncology will be an important step toward standardizing the field and promoting collaboration among healthcare professionals. As the specialty continues to evolve, efforts to reach a consensus will facilitate improved communication and knowledge sharing, ultimately enhancing patient care. Furthermore, the future of uro‐cardio‐oncology lies in the development of multidisciplinary teams that integrate expertise from various fields such as cardiology, urology, and oncology. These teams can collaborate to provide comprehensive care, devise personalized treatment strategies, and monitor patients for both cancer‐related and cardiovascular complications. Such an approach will lead to improved patient outcomes, early detection of cardiovascular problems, and tailored interventions. Finally, the future of uro‐cardio‐oncology holds great potential for advancing the understanding, diagnosis, and treatment of cardiovascular complications in patients with urological cancer. By fostering interdisciplinary collaboration, conducting further research, and establishing consensus definitions, the field can make significant strides in improving patient care and outcomes in this unique patient population.

## 9 CONCLUSIONS

There is an important relationship between cardiovascular disease and anticancer therapy. This review can serve as a foundation for a future consensus definition of uro‐cardio‐oncology. Several medication classes such as GnRH agonists, VEGFR‐TKIs, ICIs, and chemotherapeutics can cause cardiovascular complications. Gaining a comprehensive understanding of the underlying mechanisms involved will facilitate the identification of innovative therapeutic targets for cardiovascular complications. Individualized surveillance and management plans are necessary for patients with urological cancer receiving anticancer treatment. The establishment of multidisciplinary uro‐cardio‐oncology teams is crucial for improving patient outcomes.

## AUTHOR CONTRIBUTIONS

**Yi Zheng**: Conceptualization (lead); writing—original draft (lead); writing—review and editing (lead). **Ying Liu**: Writing—original draft (equal); writing—review and editing (equal). **Ziliang Chen**: Writing—review and editing (supporting). **Yunpeng Zhang**: Writing—review and editing (supporting). **Zuo Qi**: Writing—review and editing (supporting). **Ning Wu**: Writing—review and editing (supporting). **Zhiqiang Zhao**: Supervision (supporting). **Gary Tse**: Writing—review and editing (supporting). **Yong Wang**: Supervision (supporting). **Hailong Hu**: Supervision (supporting). **Yuanjie Niu**: Supervision (supporting). **Tong Liu**: Conceptualization (supporting); funding acquisition (lead); supervision (lead).

## CONFLICT OF INTEREST STATEMENT

Professor Tong Liu is the member of the *Cancer Innovation* Editorial Board. To minimize bias, he was excluded from all editorial decision‐making related to the acceptance of this article for publication. The remaining authors declare no conflict of interest.

## ETHICS STATEMENT

Not applicable.

## INFORMED CONSENT

Not applicable.

## Data Availability

Data sharing not applicable to this article as no data sets were generated or analyzed during the current study.
